# Identification of antigen-specific TCR sequences based on biological and statistical enrichment in unselected individuals

**DOI:** 10.1172/jci.insight.140028

**Published:** 2021-07-08

**Authors:** Neal P. Smith, Bert Ruiter, Yamini V. Virkud, Ang A. Tu, Brinda Monian, James J. Moon, J. Christopher Love, Wayne G. Shreffler

**Affiliations:** 1 Center for Immunology & Inflammatory Diseases, Massachusetts General Hospital, Boston, Massachusetts, USA.; 2 Harvard Medical School, Boston, Massachusetts, USA.; 3 Food Allergy Center, Massachusetts General Hospital, Boston, Massachusetts, USA.; 4 Koch Institute for Integrative Cancer Research, Massachusetts Institute of Technology, Cambridge, Massachusetts, USA.; 5 Division of Pulmonary and Critical Care Medicine, Massachusetts General Hospital, Boston, Massachusetts, USA.

**Keywords:** Immunology, T cell receptor, T cells

## Abstract

Recent advances in high-throughput T cell receptor (TCR) sequencing have allowed for new insights into the human TCR repertoire. However, methods for capturing antigen-specific repertoires remain an area of development. Here, we describe a potentially novel approach that utilizes both a biological and statistical enrichment to define putatively antigen-specific complementarity-determining region 3 (CDR3) repertoires in unselected individuals. The biological enrichment entailed FACS of in vitro antigen-activated memory CD4^+^ T cells, followed by TCRβ sequencing. The resulting TCRβ sequences were then filtered by selecting those that are statistically enriched when compared with their frequency in the autologous resting T cell compartment. Applying this method to define putatively peanut protein–specific repertoires in 27 peanut-allergic individuals resulted in a library of 7345 unique CDR3β amino acid sequences that had similar characteristics to other validated antigen-specific repertoires in terms of homology and diversity. In-depth analysis of these CDR3βs revealed 36 public sequences that demonstrated high levels of convergent recombination. In a network analysis, the public CDR3βs were shown to be core sequences with more edges than their private counterparts. This method has the potential to be applied to a wide range of T cell–mediated disorders and to yield new biomarkers and biological insights.

## Introduction

T cells are defined by their antigen-specific T cell receptor (TCR), and the collection of all TCRs in a human, which is dynamic and comprises approximately 10^10^ unique TCRs at a given time, is known as their TCR repertoire (1). TCRs are dimeric proteins, comprising an α and β chain that are both generated through the process of genomic rearrangement of germline variable (V), diversity (D), and joining (J) genes concurrent with random nucleotide insertions and deletions in the VDJ junction. This junction, known as the complementarity-determining region 3 (CDR3), interacts most closely with an epitope during antigen presentation, and is therefore the primary focus of studies aiming to elucidate the mechanisms of TCR specificity (2).

To date, ex vivo analysis of antigen-specific TCRs has largely used selection of T cells by peptide-MHC–multimer (pMHC-multimer) (e.g., tetramer) binding and FACS. Of the 78,701 listed antigen-specific TCRs in the VDJdb (https://vdjdb.cdr3.net/), 73,964 (94%) were reported to be isolated via pMHC-multimer selection (3). In addition, new methodologies aimed at defining features that confer antigen specificity have been benchmarked on various antiviral TCRs selected by pMHC tetramers (2, 4). The advantage of tetramer selection is the inherent functional validity it provides, as it labels cells specific for a single well-defined epitope. However, it comes with a major drawback in the form of a limited scope of analysis. Most immune responses are polyantigenic, and current T cell epitope mapping information on most antigens is incomplete. Additionally, pMHC tetramers can only be used with T cell donors that have genetically matched HLA alleles. Thus, tetramer selection with only known epitopes in specific genetic backgrounds will likely provide an incomplete understanding of the antigen-specific TCR repertoire and T cell–mediated immune responses.

Public TCRs defined on the basis of identical amino acid sequences across multiple individuals have been described in many contexts since the early 1990s. Their existence has been intriguing, given the vast number of possible recombination events, which suggests the prevalence of public TCRs would be much lower than what has been observed (5, 6). One major mechanism that shapes an antigen-specific public TCR repertoire is convergent recombination. Herein, selective pressure, such as that seen in response to dominant antigenic epitopes across individuals, gives rise to the presence of multiple unique nucleotide sequences producing the same “public” amino acid CDR3, due to genetic code degeneracy and homologous gene segments (7). Defining the public TCR repertoire for antigen-specific responses can help in the development of diagnostics and therapeutics in T cell–driven disorders.

Peanut allergy is a rising public health concern, and it currently affects more than 1% of the US population. Compared with other food allergies, peanut allergy is less frequently outgrown, and it presents more often with severe symptoms (8). Reactions to allergens are mediated by activation of mast cells and basophils through the high-affinity IgE receptor, occurring when receptor-bound specific IgE is cross-linked by binding to peanut allergens. Production of this high-affinity allergen-specific IgE is T cell dependent, and a peanut-specific transcriptional profile characterized by increased expression of *IL4*, *IL5*, *IL9*, and *IL13* has been observed in CD4^+^ T cell subsets from individuals with peanut allergy (9–11). Multiple forms of immunotherapy for the treatment of peanut allergy are currently in clinical trials, of which oral immunotherapy is the most common. The effects of these treatments on the peanut-specific CD4^+^ T cell response and their correlation with clinical success are active areas of investigation (12, 13).

In this work, we investigated the antigen-specific TCR repertoires of peanut-allergic individuals. We isolated peanut protein–activated and resting memory CD4^+^ T cells and sequenced the CDR3 of the TCRβ chain (CDR3β). The most enriched sequences in the peanut-activated compartment were identified as putatively peanut-specific CDR3βs (ps-CDR3s). These biologically and statistically enriched sequences exhibited properties associated with antigen-specific populations, such as increased homology, decreased diversity, and instances of convergent recombination. Within our pool of ps-CDR3s, we found a subset of sequences enriched in multiple subjects (i.e., public ps-CDR3s), suggesting the existence of public T cell responses among peanut-allergic individuals with diverse HLA genotypes. This work describes a potentially novel method to study antigen-specific TCR repertoires without restriction to known T cell epitopes or the availability of matched MHC reagents. This method is potentially applicable to studies of various T cell–mediated diseases beyond the scope of allergy.

## Results

### Selection of ps-CDR3s.

**Figure 1:**
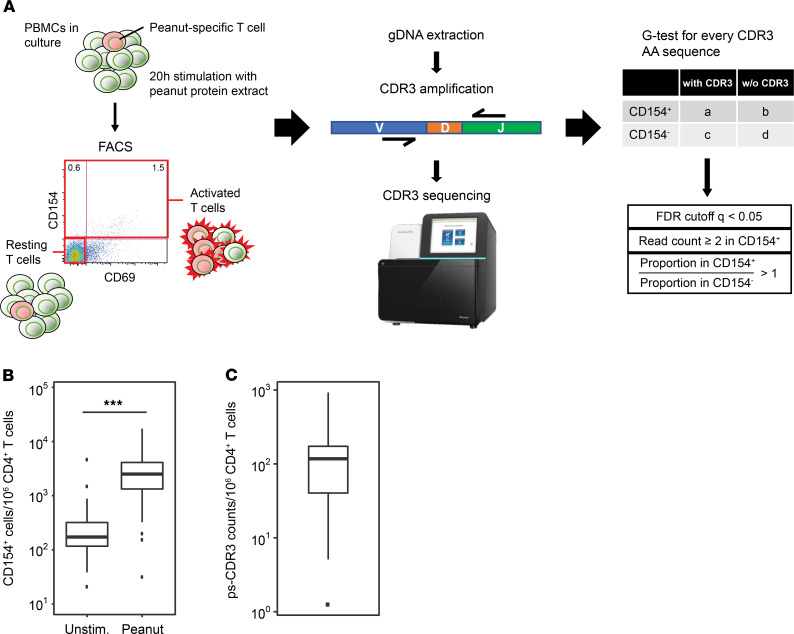
Selection of putatively peanut-specific CDR3β sequences. (**A**) A general schema for the selection of putatively peanut-specific CDR3β sequences (ps-CDR3s) (see Methods). (**B**) The frequency of CD154^+^ T cells per million CD4^+^ T cells in peanut protein–stimulated PBMC cultures is higher than in unstimulated cultures (*n =* 27, ***P < 0.001, Wilcoxon matched-pairs signed-rank test). (**C**) The frequency of ps-CDR3s per million CD4^+^ T cells in peanut protein–stimulated cultures (*n =* 27).

With the goal of enriching for antigen-specific memory CD4^+^ T cells, PBMCs from 27 peanut-allergic individuals were cultured for 20 hours with a peanut protein extract. Demographic and serological data from the participants in this study can be found in Table 1. The activation marker CD154 (CD40L) was used to discriminate between peanut-activated (CD154^+^) and resting (CD154^–^CD69^–^) memory T cells, and TCR CDR3β sequences were generated from both populations (Figure 1A; refs. 10, 14; Supplemental Figure 1; and Supplemental Table 1; supplemental material available online with this article; https://doi.org/10.1172/jci.insight.140028DS1). Flow cytometric analysis revealed that stimulation with peanut protein increased the frequency of CD154-expressing CD4^+^ memory T cells as compared with unstimulated cultures (median CD154^+^ cells per million CD4^+^ T cells, 2504 [IQR = 1328, 4105] stimulated versus 172 [IQR = 117, 319] unstimulated; *P <* 0.0001; Figure 1B). To filter out sequences that were likely to be present in the CD154^+^ population due to bystander activation, we developed a statistical enrichment strategy to define ps-CDR3s. First, a *G* test of independence was applied to all CDR3β sequences in the peanut-activated compartment, and adjusted *P* values (*q* values) were generated based on a FDR of *q* < 0.05. Sequences were further filtered to include only those with read counts ≥2 in the activated compartment and those with a higher proportion in the activated compartment than in the resting compartment. This approach defined 7345 of the 53,205 unique amino acid sequences in the peanut-activated compartment (14%) across our 27 peanut-allergic individuals as ps-CDR3s, which met all 3 selection criteria (see Methods). While the vast majority (*n =* 7309) of the ps-CDR3s were private, 36 unique amino acid sequences (0.49% of ps-CDR3s) were public, being enriched in more than 1 individual (i.e., public). Of these public sequences, 33 were found in only 2 individuals. However, 1 ps-CDR3 (CASSFRFLSGRALNEQFF) was statistically enriched in 8 of the 27 individuals, suggesting strong selective pressure for entrance of this sequence into the peanut-specific memory T cell repertoire (Supplemental Figure 2). The CDR3β amino acid and nucleotide sequences that met our ps-CDR3 enrichment criteria only represented a minor fraction of the entire CDR3β pool from peanut-activated T cells (amino acid, median 14.1%; nucleotide, median 14.5%), indicating a high level of stringency with this statistical approach. The median frequency of ps-CDR3s was 117 per million CD4^+^ T cells (IQR = 46.8, 173.15); this corresponds well to published frequencies of whole allergen–specific T cells in allergic individuals, suggesting the level of stringency is appropriate (Figure 1C and refs. 11, 15, 16).

We compared this strategy for TCR enrichment to the established approach of selecting antigen-specific TCRs by pMHC tetramer binding. PBMCs from 1 of the 27 individuals were cultured with the major peanut allergen Ara h 1 for 20 hours to isolate CD154^+^ and CD154^–^ T cells by FACS or for 2 weeks to expand the Ara h 1–specific T cell population and isolate Ara h 1 (amino acid 415–425) tetramer^+^ and tetramer^–^ T cells (Supplemental Figure 3A). TCR CDR3β sequences were generated, and our method of statistical enrichment was applied to both populations, comparing CD154^+^ to CD154^–^ CDR3βs and tetramer^+^ to tetramer^–^ CDR3βs respectively, to filter out bystander-activated clones or those present due to nonspecific binding of tetramer. We selected 458 ps-CDR3s from the CD154^+^ population (accounting for 34.1% of total CD154^+^ reads, and 17.9% of unique CD154^+^ CDR3βs) and 175 ps-CDR3s from the tetramer^+^ population (82.2% of total tetramer^+^ reads, 17.3% of unique tetramer^+^ CDR3βs), and detected 11 clones that were present in both populations (Supplemental Figure 3B). Interestingly, these 11 ps-CDR3s were among the most expanded clones in the tetramer^+^ population, indicating that our approach captures the most dominant peanut-specific TCRs (Supplemental Figure 3C). Moreover, the relatively low frequency of tetramer^+^ clones present in the CD154^+^ population (11 of 458, or 2.4%) highlights the capacity of this strategy to identify ps-CDR3s beyond those specific for the single epitope used in the pMHC tetramer.

### ps-CDR3s are more similar and less diverse than unselected CDR3βs from both activated and resting T cells.

**Figure 2:**
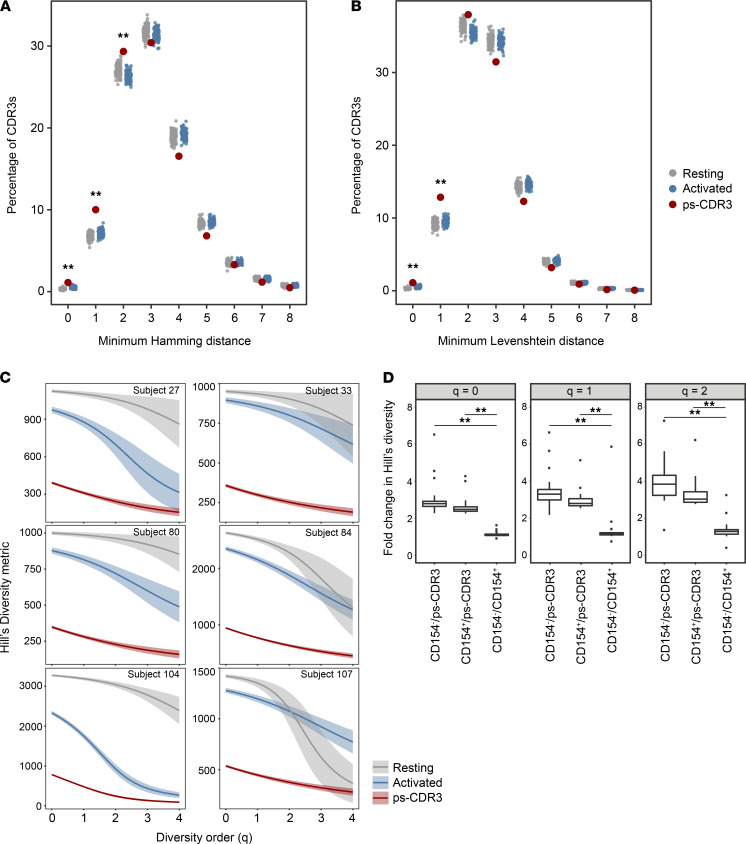
Putatively peanut-specific CDR3β sequences demonstrate increased similarity and decreased diversity when compared with total activated and resting CDR3βs. (**A** and **B**) Minimum Hamming and Levenshtein distance of putatively peanut-specific CDR3β sequences (ps-CDR3s) compared with 100 equal-sized random resamplings of total activated and resting CDR3βs (**P < 0.01, ps-CDR3 vs. resting and ps-CDR3 vs. activated, Fisher’s exact test). (**C**) Smoothed Hill’s diversity curve at diversity orders 0–4 for 6 individuals. Diversity of ps-CDR3s was significantly lower than that of activated and resting CDR3βs at all diversity orders (*P <* 0.01, empirical cumulative distribution function of bootstrap delta distribution, see Methods). (**D**) Fold change of Hill’s diversity metric of resting/ps-CDR3s, activated/ps-CDR3s, and resting/activated CDR3βs. Distributions represent values from 25 individuals with >30 unique ps-CDR3s (**P < 0.01, Wilcoxon matched-pairs signed-rank test).

To assess the efficacy of our statistical enrichment method for selecting those sequences most likely to be peanut specific, we examined the similarity of the ps-CDR3s using Hamming and Levenshtein distances. For each ps-CDR3, the minimum Hamming and Levenshtein distances were measured by determining the minimum number of amino acid differences compared with its next closest ps-CDR3. As a control, the minimum Hamming and Levenshtein distances of equal-sized random repeated samplings of resting (CD154^–^) and activated (CD154^+^) CDR3βs were also measured (Figure 2, A and B). There were significantly more sequences with a minimum Hamming distance of 0–2 and Levenshtein distance of 0–1 in the ps-CDR3s than in either the activated or resting CDR3βs (*P <* 0.01), indicating enhanced similarity among the statistically enriched sequences. Importantly, these metrics showed no differences between unselected activated versus resting CDR3βs, supporting our hypothesis that there is a substantial number of nonpeanut-specific sequences in the CD154^+^ population and that our statistical enrichment strategy is effective in removing them.

We performed uniform resampling of autologous sets of ps-CDR3s and activated and resting CDR3β sequences to measure Hill’s diversity index across different diversity orders to capture both the true richness (low *q* value) and abundance (high *q* value) of the samples (Figure 2C and ref. 17). For the 25 individuals with more than 30 ps-CDR3s, the general diversity index was significantly lower for the ps-CDR3s than for either the activated or resting sequences at diversity orders 0–2 (*P <* 0.01). Consistent with biological enrichment, the diversity of the activated sequences was significantly lower than that of the resting samples at diversity orders 0–2 for 22 of the 25 individuals (*P <* 0.01). Furthermore, the difference in diversity between the ps-CDR3s and either the activated or resting CDR3βs was significantly greater than the difference observed between the activated and resting CDR3βs (*P <* 0.001), emphasizing the utility in our statistical filtering (Figure 2D).

### Public ps-CDR3s are closer to germline and show evidence of convergent recombination.

**Figure 3:**
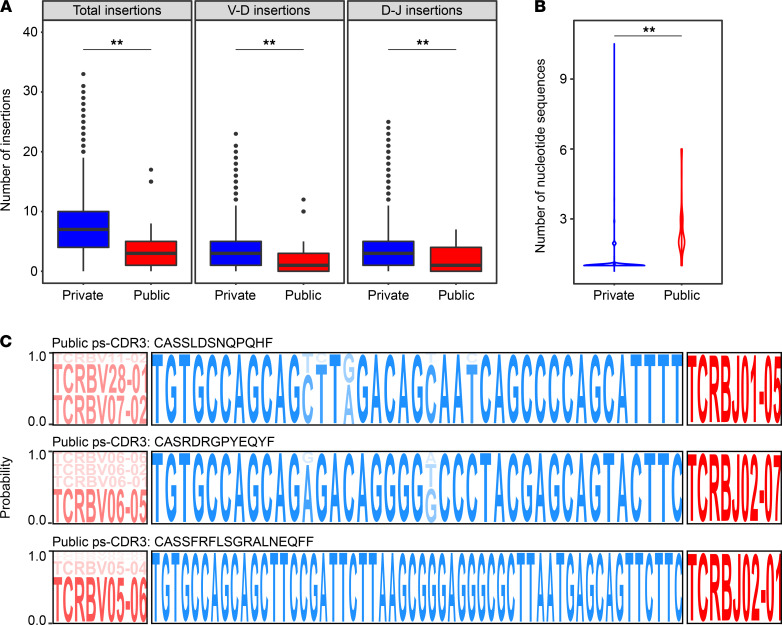
Public putatively peanut-specific CDR3β sequences are characterized by being closer to germline and exhibiting convergent recombination. (**A**) Public putatively peanut-specific CDR3β sequences (ps-CDR3s) are closer to germline than private ps-CDR3s. Distribution of total, V-D, and D-J insertions of public and private ps-CDR3s (**P < 0.01, Mann-Whitney *U* test). (**B**) Public ps-CDR3s demonstrate more convergent recombination than private ps-CDR3s. Violin plots represent the number of unique nucleotide sequences responsible for each unique public and private ps-CDR3 (**P < 0.01, Mann-Whitney *U* test). (**C**) Convergence of public ps-CDR3s occurs in both germline (V gene) and nongermline (CDR3β) regions. Logo plots represent the probability of either specific gene segments (red) or CDR3β nucleotides (blue) to be encoding for their corresponding amino acid sequence.

Antigen-specific public TCRs from individuals with different HLA genotypes can provide valuable insights into shared immune responses within a diverse population. For this reason, we wanted to further examine the characteristics of our public ps-CDR3s. We observed that the public ps-CDR3s contained fewer total N-nucleotide insertions than the private ps-CDR3s (median public, 3 insertions; median private, 7 insertions; *P <* 0.01). Similar patterns were observed when looking specifically at V-D insertions (median public, 1 insertion; median private, 3 insertions; *P <* 0.01) and D-J insertions (median public, 1 insertion; median private, 3 insertions; *P <* 0.01), which is consistent with prior literature demonstrating that public TCRs are closer to germline (Figure 3A and refs. 7, 18–20).

Convergent recombination of TCRs (i.e., multiple TCR nucleotide sequences translating to the same amino acid sequence) is a characteristic of antigenic selection (7). We searched the ps-CDR3s for this phenomenon and observed that convergence occurred in 467 of 7345 unique ps-CDR3 amino acid sequences (6.4%). Interestingly, convergence was very common among the public ps-CDR3s, occurring in 33 of 36 public amino acid sequences (91.7%), which suggests a strong selective pressure for these sequences’ entrance into the peanut-specific memory T cell pool. Additionally, the number of nucleotide sequences encoding each public ps-CDR3 was significantly higher than the number for each private ps-CDR3 (median public, 2 sequences; median private, 1 sequence; *P <* 0.001; Figure 3B). Analysis of the 3 most convergent public ps-CDR3s (≥5 unique nucleotide sequences) demonstrated convergence derived from germline (V gene) and nongermline (N-nucleotide insertions) changes (Figure 3C).

To obtain full TCR sequences of the public ps-CDR3s, PBMCs from 12 of the 27 peanut-allergic individuals from the initial analysis were stimulated with peanut protein extract for 20 hours and single-cell TCRα and TCRβ capture and sequencing were performed by Seq-Well on FACS-sorted peanut-activated CD154^+^ memory T cells (21). Of the 36 public ps-CDR3 TCRβ sequences, 15 were found in at least 1 cell (41.7%), with 10 having matched TCRα and TCRβ sequences (27.8%; Table 2).

### Analysis of ps-CDR3 amino acid sequences reveals public, dominant motifs.

**Figure 4:**
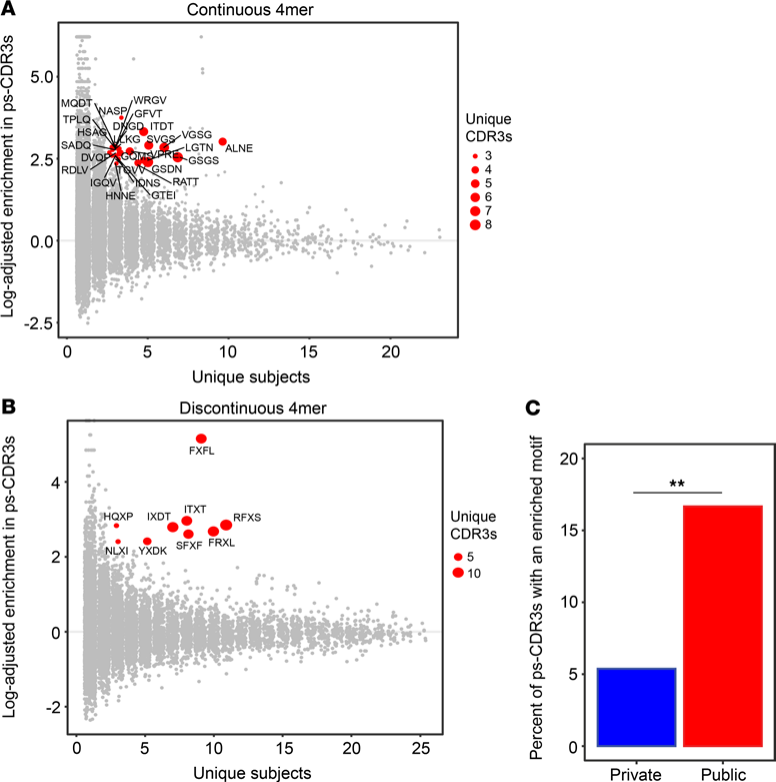
Putatively peanut-specific CDR3β sequences contain public, dominant motifs. (**A** and **B**) The putatively peanut-specific CDR3β sequences (ps-CDR3s) contain shared, enriched motifs as compared with resting CDR3βs. Red points represent the most dominant motifs found to be ≥10-fold enriched in ps-CDR3s as compared with resting CDR3βs and present in ≥3 unique ps-CDR3s derived from ≥3 individuals. (**C**) Public ps-CDR3s are more likely to have a dominant motif than private ps-CDR3s (**P < 0.01, Fisher’s exact test).

Given that the ps-CDR3 sequences demonstrated enhanced overall similarity, a motif analysis was performed according to Glanville et al. (2) to find dominant patterns among the putatively peanut-specific pool of sequences (Supplemental Figure 4A). Briefly, for all ps-CDR3s, the region most likely to be in contact with an antigenic peptide was broken into continuous 3-mer, 4-mer, and 5-mer amino acid sequences. In addition, discontinuous 4-mers and 5-mers were generated to allow for the detection of motifs with gaps. N-mers that were at least 10-fold enriched in the ps-CDR3s as compared with the resting sequences and found in at least 3 unique ps-CDR3s from at least 3 individuals were considered dominant motifs (Figure 4, A and B, and Supplemental Figure 4, B–D). These criteria were met by 148 of the 104,520 unique n-mer sequences (0.14%), and the proportion of dominant motifs in the total motif pool was fairly similar for each n-mer size (0.06%–0.16%; Table 3). With these strict criteria, 399 of the 7345 ps-CDR3s (5.4%) contained at least 1 dominant motif. These motifs likely represent sequences specific for the most immunogenic T cell epitopes in peanut protein. Indeed, there were significantly more public ps-CDR3s (16.6%) than private ps-CDR3s (5.3%) that contained a dominant motif, supporting the hypothesis that these motifs are associated with the most common epitopes (*P <* 0.05; Figure 4C).

### Network analysis confirms that public ps-CDR3s are core sequences.

**Figure 5:**
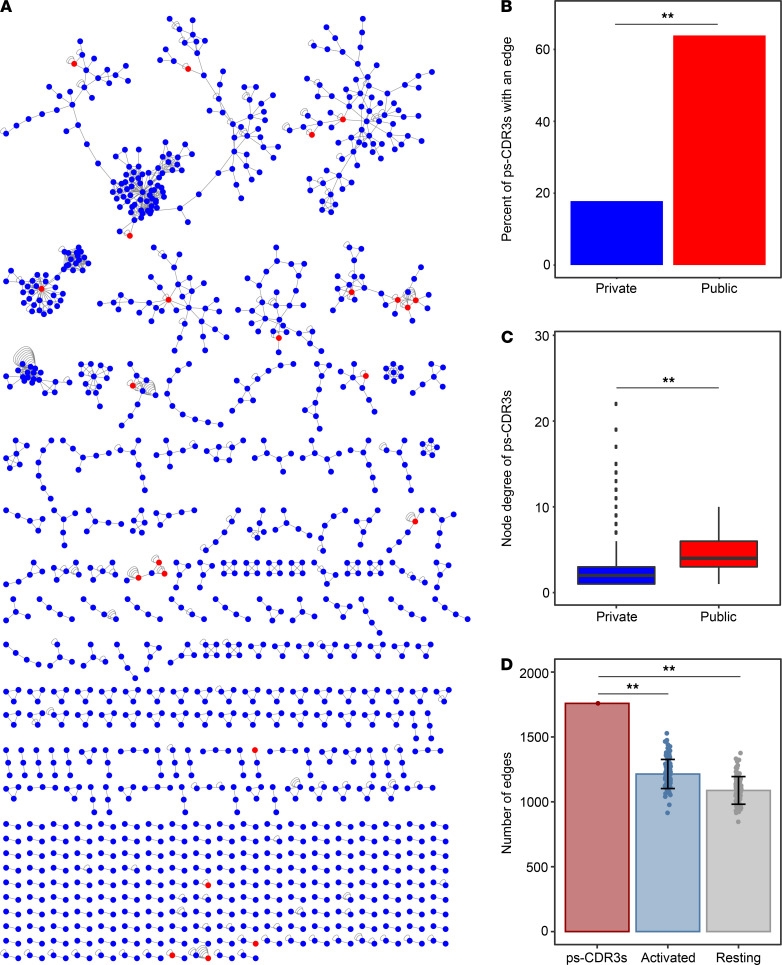
Network analysis reveals that public putatively peanut-specific CDR3β sequences are core sequences with more structure than activated and resting CDR3βs. (**A**) Network analysis of putatively peanut-specific CDR3β sequences (ps-CDR3s), where each node represents a unique ps-CDR3 amino acid sequence. Edges were created between the nodes if these were within a Levenshtein distance of 1 or if the nodes shared an enriched motif. Self-edges were created on nodes to represent additional unique nucleotide sequences encoding the same amino acid sequence (i.e., convergence). Blue nodes represent private ps-CDR3s, and red nodes represent public ps-CDR3s. (**B**) The percentage of public ps-CDR3s with an edge to another ps-CDR3 (as shown in **A**) was higher than that of private ps-CDR3s (**P < 0.01, Fisher’s exact test). (**C**) The overall node degree (number of edges per node) of public ps-CDR3s was higher than that of private ps-CDR3s. Box plots represent distribution of the node degree of all private (blue) and public (red) ps-CDR3s in the graph (**P < 0.01, Mann-Whitney *U* test). (**D**) The number of total edges created among the ps-CDR3s compared with the median number of edges created among 100 equal-sized random resamplings of total activated and resting CDR3βs. Error bars represent standard deviation (**P < 0.01, *Z* test).

Utilizing the measured metrics of similarity, the dominant motifs, and evidence of convergent recombination, we performed a network analysis to group highly homologous sequences (Figure 5A). Similar to previously published approaches (2, 22, 23), each ps-CDR3 amino acid sequence was treated as a node and an edge was created between nodes if the ps-CDR3s were within a Levenshtein distance of 1 or shared a dominant motif. In addition, self-edges were created to represent additional nucleotide sequences for a corresponding amino acid sequence. Overall, there were 1759 edges created among 1324 ps-CDR3s. Interestingly, the majority (*n =* 23; 63.9%) of public ps-CDR3s were present in the network, whereas a much smaller fraction of private ps-CDR3s (*n =* 1301; 17.7%) were found in the graph (*P <* 0.001; Figure 5B). Moreover, the node degree (the number of edges per node) of the public sequences was higher than that of the private sequences (public median node degree, 4; private median node degree, 2; *P <* 0.001; Figure 5C). To assess the amount of structure observed among the ps-CDR3s, we compared the number of edges in the ps-CDR3 graph to the median number of edges created when performing the same network analysis on equal-sized random repeated samplings of activated or resting CDR3βs. Indeed, there were significantly more edges created between the ps-CDR3s than between all activated (CD154^+^) CDR3βs (median 1234 edges) or resting (CD154^–^) CDR3βs (median 1091 edges) (*P <* 0.001; Figure 5D). We hypothesized that specific clusters of CDR3βs would be associated with individual HLA genotypes, which are shown in Supplemental Table 2, but no correlations could be found. Taken together, these data suggest that public ps-CDR3s represent “core” sequences that likely bind dominant T cell epitopes in peanut protein, which are recognized by multiple peanut-allergic individuals.

### The frequency of ps-CDR3s is highest in the Th17 subset, but the clonal expansion of ps-CDR3s is highest in the Th2 subset.

**Figure 6:**
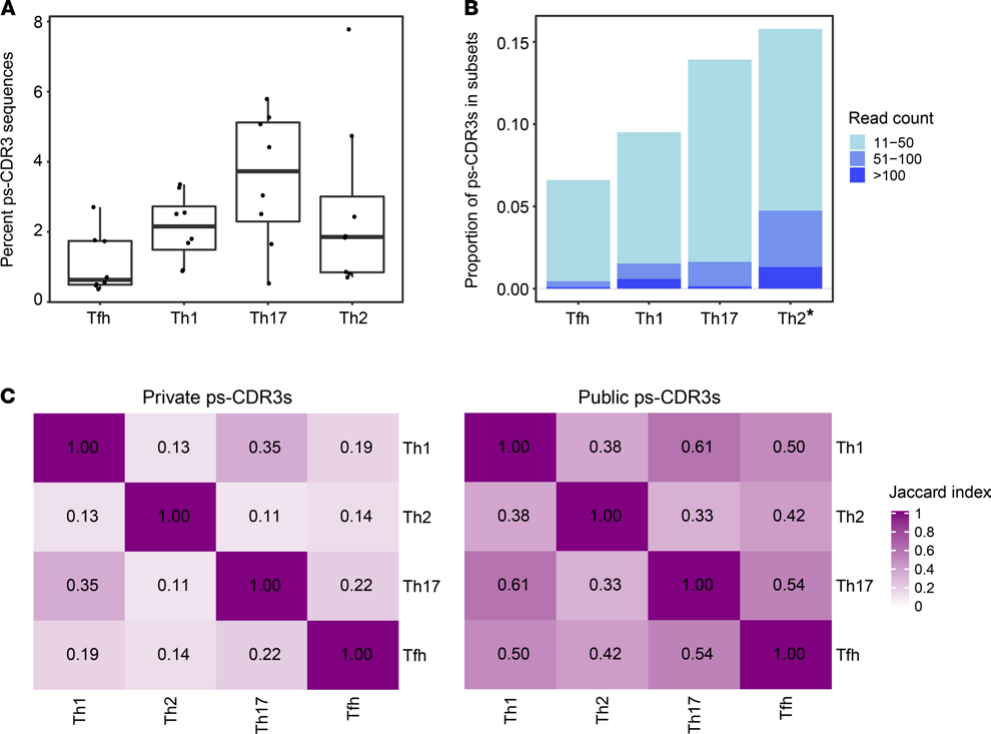
The frequency of putatively peanut-specific CDR3β sequences is highest in the Th17 subset, but the clonal expansion of putatively peanut-specific CDR3β sequences is highest in the Th2 subset. (**A**) The percentage of total read counts corresponding to a putatively peanut-specific CDR3β sequence (ps-CDR3) was highest in the Th17 subset (*n =* 8). (**B**) Th2-derived ps-CDR3s were more clonally expanded than ps-CDR3s in the other subsets. Bar represents the proportion of ps-CDR3s in a given subset based on their read count (*P < 0.01, logistic regression). (**C**) Jaccard index showing the degree of overlap in public and private ps-CDR3s between the T cell subsets.

To better understand the phenotypes of the CD4^+^ T cells from which ps-CDR3s were derived, TCRβ sequencing was performed on bulk Th1 (CXCR3^+^CCR5^+^), Th2 (CRTH2^+^CCR4^+^), Th17 (CD161^+^CCR6^+^) and T follicular helper (Tfh) (CXCR5^+^) cells from 8 of the 27 individuals used in the initial analysis (Supplemental Figure 5 and Supplemental Table 1). These bulk TCRβ sequences were then probed for ps-CDR3 amino acid sequences derived from the corresponding individuals, and the proportion of ps-CDR3 reads was observed to be highest in the Th17 subset (Figure 6A). However, ps-CDR3s detected in the Th2 subset showed a higher level of clonal expansion than ps-CDR3s in the other subsets, indicating that the most dominant peanut-specific T cell clones have a Th2 phenotype (*P <* 0.01; Figure 6B). Although ps-CDR3s were predominantly detected within a single T cell subset, we also observed shared sequences across the subsets, and these were more common among the public ps-CDR3s than private ps-CDR3s. In addition, shared sequences were most often found between the Th1 and Th17 phenotypes (Figure 6C). These data suggest either shared epitope recognition by T cells from these subsets or plasticity between Th1 and Th17 cell states, supporting recently published data on colonic T helper subsets in the gut (24).

## Discussion

Selection of antigen-specific T cells based on their ability to bind an MHC peptide complex (pMHC-multimer selection) is a widely used technique. This method, however, depends on a priori knowledge of T cell epitopes in individuals with specific HLA backgrounds and is, therefore, limited in scope. Here, we expand upon a method not constrained by these limitations, utilizing a biological enrichment and augmenting it with a statistical enrichment to isolate likely antigen-specific CDR3βs that are not derived from bystander-activated T cells. This approach generated a library of ps-CDR3s that exhibited properties of an antigen-specific pool in terms of diversity, homology, and convergence. Using these features in a network analysis to cluster similar sequences, we were able to show that public ps-CDR3s tended to have more neighbors (i.e., were more similar) than private ps-CDR3s, which corresponds with observations made by Madi et al. (23). When using the ps-CDR3s to probe the Th cell subset repertoires from a selection of the peanut-allergic individuals, we found that ps-CDR3s were most frequently present within the Th17 subset, but those ps-CDR3s within the Th2 subset were most clonally expanded. These data substantiate previous observations from our group, as we found that peanut-activated memory CD4^+^ T cells from peanut-allergic patients highly express both Th17- and Th2-related genes. However, only Th2-related gene expression and cytokine production distinguished patients with high clinical sensitivity to peanut from those with low sensitivity (10).

The use of CD154 as a marker to isolate antigen-activated T cells was first described in 2005, when Frentsch et al. demonstrated the utility of this method with an array of microbial and allergenic antigens (25). Since then, numerous other groups have successfully applied this method with allergens and pathogen-derived proteins to better describe the phenotypes of antigen-specific CD4^+^ T cell populations (11, 14, 26–29). However, utilizing this methodology to better characterize antigen-specific TCR repertoires is in its infancy. Glanville et al. single-cell sorted and sequenced the TCRs of CD154^+^ T cells activated with an *M*. *tuberculosis* lysate and successfully validated antigen-specific motifs with an in vitro binding assay, demonstrating that CD154 upregulation is a valid method to study antigen-specific repertoires (2). Given the concern of bystander activation of nonantigen-specific T cells, we chose to use the CD154 upregulation assay in conjunction with a statistical enrichment approach to define our library of ps-CDR3s. This statistical enrichment has the distinct advantage of utilizing autologous resting memory TCR repertoires as a reference when selecting the ps-CDR3s, therefore controlling for the presence of clones in the CD154^+^ compartment due to bystander activation of common clones. The ps-CDR3s exhibited characteristics associated with antigen-specific TCR pools, such as increased similarity and decreased diversity, when compared with both resting as well as total activated CDR3βs. These findings emphasize the utility of applying the biological (CD154 upregulation) and statistical (*G* test of independence) enrichments in combination when determining the most relevant TCRs.

The application of this method for defining antigen-specific ps-CDR3s has the potential to answer a wide array of biological questions. As described here, antigen-specific ps-CDR3 libraries can be used to probe phenotypically defined subsets of CD4^+^ T cells to better understand the phenotype and physiology of antigen-specific T cells. Recently, our group has successfully used a similar approach to identify differences in the peanut-specific CD4^+^ T cell repertoire and phenotypes between peanut-allergic individuals with high and low clinical sensitivity (10). We found that high clinical sensitivity correlates with an expanded and more diverse peanut-specific effector T cell population. Our strategy could also be extended to track antigen-specific ps-CDR3s across a time course or therapy. Examining the clones that expand or contract in response to an intervention could lead to insights regarding efficacy or susceptibility to side effects.

A major advantage of our approach is that it is unbiased in terms of MHC restriction, representing the complete CD4^+^ T cell response to one or multiple antigenic proteins, which is unlikely to be captured with pMHC-multimer–based sorting. In a previous study, Ryan et al. used a pMHC-dextramer approach to select Ara h 2–specific T cells from peanut-allergic individuals with a defined HLA background (HLA-DRB1*1501, *HLA-DRB4*; ref. 12). While this method was successful in identifying phenotypic changes in Ara h 2–specific T cells over the course of peanut oral immunotherapy, the authors were only able to define 13 Ara h 2–specific TCRs, providing limited insights into the peanut-specific repertoire. Of note, we did not find any of the 13 reported Ara h 2–specific TCRs within our ps-CDR3s. In the present study, we observed that the most expanded CDR3β sequences captured by using an Ara h 1 (amino acid 415–425) pMHC tetramer were also present among ps-CDR3s from Ara h 1–activated CD154^+^ T cells of the same patient (Supplemental Figure 3). These data indicate that our approach, based on biological and statistical enrichment, detects the most relevant CDR3βs specific for known T cell epitopes. Furthermore, the ps-CDR3s from Ara h 1–activated T cells included hundreds of additional antigen-enriched sequences, suggesting our epitope-agnostic approach is capturing more of the antigen-specific repertoire.

Using a method agnostic to both HLA genotype and known epitopes can help to better define the TCR landscape of a given immune response. For example, our study enabled us to describe the public nature of 36 unique antigen-specific CDR3βs. This would have been difficult to capture with methods based on pMHC multimers, given the very low numbers of antigen-specific T cells detected by these methods and the sparsity of these T cells in the overall T cell repertoire. The propensity of the public ps-CDR3s to demonstrate convergent recombination further exemplifies the utility of our methodology, as convergence suggests antigenic selection (7, 30, 31). Through the use of single-cell sequencing technologies, we were able to elucidate the matching TCRα and TCRβ chains for 10 of the public ps-CDR3s, demonstrating that these two methodologies in conjunction can help resolve the complete sequences that are most important for the development of diagnostics and/or therapeutics.

In sum, by using a combination of biological and statistical enrichment of CDR3βs from antigen-activated CD4^+^ T cells, we were able to identify private and public CDR3βs that show evidence of antigenic selection. This method holds promise for application in a wide range of T cell–mediated disorders, as it effectively interrogates the antigen-specific TCR repertoire in unselected individuals, and potentially yields biomarkers for disease state and response to various treatments.

## Methods

### Participants.

The individuals included in this study participated in a peanut oral immunotherapy trial (NCT01750879) conducted at the Food Allergy Center at Massachusetts General Hospital. Criteria screened for the study included having a previous diagnosis of peanut allergy, a history of peanut-induced reactions consistent with immediate hypersensitivity, and peanut-specific serum IgE levels greater than 5 kU/L (ImmunoCAP; Thermo Fisher). Individuals who met these criteria underwent a double-blind placebo-controlled food challenge to confirm peanut allergy. Increasing peanut protein doses were administered every 20 minutes up to a maximum dose of 300 mg according to the following schedule: 3, 10, 30, 100, and 300 mg. All 27 individuals included in this study had an objective allergic reaction during the food challenge.

### Cell culture and sorting of peanut-activated and resting memory CD4^+^ T cells.

Patient blood samples were collected at baseline (before the food challenge and the start of peanut oral immunotherapy), and PBMCs were isolated by means of density gradient centrifugation (Ficoll-Paque Plus; GE Healthcare). Fresh PBMCs were cultured in AIM V medium (Gibco) for 20 hours at a density of 5 × 10^6^ cells in 1 mL medium per well in 24-well plates and were left unstimulated or cultured with 100 μg/mL peanut protein extract (15 × 10^6^ PBMCs per variable). The peanut extract was prepared by agitating defatted peanut flour (Golden Peanut and Tree Nuts) with PBS, centrifugation, and sterile filtering. PE-conjugated anti-CD154 (clone TRAP1; BD Biosciences) was added to the cultures (20 μL/well) for the last 3 hours. After harvesting, the cells were labeled with AF700-conjugated anti-CD3 (clone UCHT1), APC-Cy7–conjugated anti-CD4 (RPA-T4), FITC–conjugated anti-CD45RA (HI100), and PE-conjugated anti-CD154 (all from BD Biosciences); AF647-conjugated anti-CD69 (FN50; BioLegend); and Live/Dead Fixable Violet stain (L34955; Thermo Fisher). Live CD3^+^CD4^+^CD45RA^–^ CD154^+^ activated T cells and Live CD3^+^CD4^+^CD45RA^–^CD154^–^CD69^–^ resting T cells were sorted with a FACSAria II instrument (BD Biosciences). Sorted T cells were lysed in Buffer RLT Plus (Qiagen) with 1% β-mercaptoethanol (MilliporeSigma) and stored at –80°C, before total RNA and genomic DNA were isolated using the AllPrep DNA/RNA Micro Kit (Qiagen).

### Expansion of Ara h 1–specific CD4^+^ T cells and sorting of pMHC tetramer^+^ T cells.

Cryopreserved PBMCs from 1 of the 27 individuals (patient 107) were thawed and cultured in RPMI 1640 supplemental with 2 mM Glutamax (both from Gibco), 10% human serum (MilliporeSigma), and 100 U/ml penicillin and 100 μg/ml streptomycin (Thermo Fisher) (complete RPMI), with 50 μg/ml natural Ara h 1 (Indoor Biotechnologies), at a density of 6 × 10^6^ cells in 1 ml medium per well in 24-well plates. Complete RPMI + 10 U/mL IL-2 (R&D Systems) was added after 5 days, and cells were cultured for a total of 14 days to expand Ara h 1–specific CD4^+^ T cells. After harvesting, memory CD4^+^ T cells were isolated with the EasySep human memory CD4^+^ T cell enrichment kit (Stemcell Technologies) and labeled with APC-conjugated Ara h 1 (DRB1*03:01, amino acid 415–425) tetramer (32) (made in-house), at a concentration of 10 nM for 1 hour at room temperature. After washing off excess tetramer, the cells were labeled with BUV395-conjugated anti-CD3 (clone UCHT1; BD Biosciences), APC-Cy7–conjugated anti-CD4, FITC-conjugated anti-CD45RA, and Live/Dead Fixable Blue stain (L23105; Thermo Fisher) for 30 minutes at 4°C. Live CD3^+^CD4^+^CD45RA^–^ tetramer^+^ and tetramer^–^ T cells were sorted with a FACSAria Fusion instrument (BD Biosciences), and genomic DNA was isolated using the AllPrep DNA/RNA Micro Kit.

### TCRβ sequencing.

Genomic DNA was used to amplify and sequence CDR3 regions (immunoSEQ assay; Adaptive Biotechnologies). The immunoSEQ approach generates an 87–base-pair fragment capable of identifying the VDJ region spanning each unique CDR3β. Amplicons were sequenced using the Illumina NextSeq platform. Using a baseline developed from a suite of synthetic templates, primer concentrations and computational corrections were used to correct for the primer bias common to multiplex PCR reactions. Raw sequence data were filtered on the basis of TCRβ V, D, and J gene definitions provided by the IMGT database (http://www.imgt.org/) and binned using a modified nearest-neighbor algorithm to merge closely related sequences and remove both PCR and sequencing errors.

### Selection of ps-CDR3s.

TCRβ-sequencing data from activated (CD154^+^) and resting (CD154^–^CD69^–^) memory CD4^+^ T cell samples of all 27 peanut-allergic individuals were parsed using the *tcR* package (version 2.2.3) in R (33). We applied a statistical method to define ps-CDR3s, i.e., sequences that are likely to be antigen specific and not detected due to bystander activation. This methodology’s workflow starts with performing a *G* test of independence on every unique CDR3β found in an individual’s activated CD154^+^ T cell sample to determine if the proportion of a given sequence is higher in the activated CDR3βs than the resting CDR3βs. A *G* test (also known as a likelihood ratio test) can be described by the following formula:

(Equation 1)
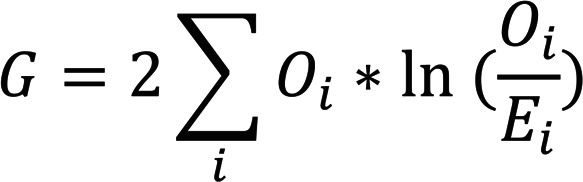


where *O_i_* represents the observed read count of a given CDR3β in a particular population (activated or resting) and *E_i_* denotes the expected count based on the proportion of this CDR3β in the entire memory T cell population (pooled activated and resting CDR3βs). With the *G* value for each CDR3β, the probability that the proportion of a CDR3β in the activated compartment is derived by chance from the proportion in the resting compartment can be calculated. All *G* tests were calculated using the *GTest* function from the R package *Desctools* (version 0.99.34; ref. 34). Given the sizable number of CDR3βs that were analyzed from each individual’s data, a FDR correction was used to generate *q* values for each sequence, and those that met a cutoff of *q* < 0.05 were considered significant for this study. To enhance the stringency and reduce type 1 error, we also filtered out those CDR3βs with a read count of only 1 in the activated compartment (*n =* 24,520, 46%) and those with a proportion in the activated compartment of less than that in the resting compartment (*n =* 4, 0.008%). All sequences selected after filtering were deemed ps-CDR3s.

### Minimum Hamming and Levenshtein distances.

To determine global levels of similarity, the minimum Hamming distance (number of amino acid differences among CDR3βs of same length) and minimum Levenshtein distance (minimum number of insertions/deletions/substitutions between CDR3βs) of each ps-CDR3 against all other ps-CDR3s was determined using R (version 3.5.1) with the package *stringdist* (version 0.9.5.1). The percentage of ps-CDR3s at each minimum Hamming or Levenshtein distance was calculated. As a control, the minimum Hamming and Levenshtein distances of 100 equal-sized random resamplings of CDR3βs from the total activated and resting T cell pools were determined. The median percentage of sequences at each minimum Hamming and Levenshtein distance for the 100 resamplings was calculated.

### Diversity measurements.

Diversity curves that measured Hill’s diversity metric across diversity orders 0–4 were created using the R package *alakazam* (version 0.3.0) with the *alphaDiversity* function (17). Hill’s diversity metric can be described by the following formula:

(Equation 2)
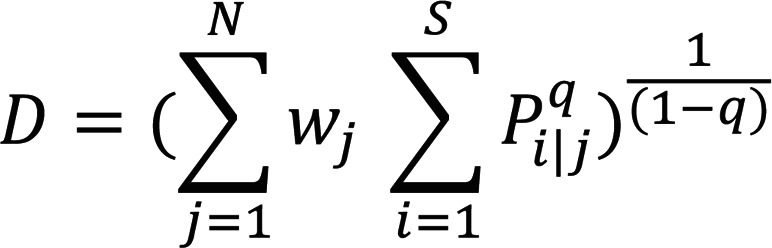


where *i* represents a unique CDR3β, *j* the sample size, *P_i|j_* the proportional abundance of the *i^th^* CDR3β in the *j^th^* sample, *W_j_* the proportional abundance of the *j^th^* sample relative to the entire data set, and *q* represents a tuning parameter. *q* controls the influence of abundant over rare species on the metric (as *q* increases, the abundance of dominant sequences is more heavily weighted). Therefore, examining Hill’s diversity metric across different values of *q* captures both the richness and abundance of species. The fold change of Hill’s diversity metric of all pairwise combinations of ps-CDR3s, total activated CDR3βs, and resting CDR3βs was determined and the differences in these distributions were compared.

### Paired single-cell TCRα/β sequencing.

In parallel experiments, peanut protein–activated CD154^+^ memory CD4^+^ T cells from 12 of the 27 individuals were processed for single-cell RNA sequencing using the Seq-Well platform as previously described (35). A portion of each cDNA library was reserved for paired TCRα/β enrichment. Paired TCR sequencing was performed according to Tu et al. (21). Briefly, following cDNA amplification, biotinylated capture probes for human TRAC and TRBC regions were annealed to cDNA. Magnetic streptavidin beads were used to enrich the bound TCR sequences, which were then further amplified using human V-region primers and prepared for sequencing using Nextera sequencing handles. Libraries were sequenced on an Illumina MiSeq using 150 bp length reads.

TCR-sequencing reads were preprocessed according to Tu et al (21). In short, reads were mapped to TCRV and TCRJ IMGT reference sequences via IgBlast, and V and J calls with “strong plurality” (wherein the ratios of the most frequent V and J calls to the second most frequent calls were at least 0.6) were retained. CDR3 sequences were called by identifying the 104-cysteine and 118-phenylalanine according to IMGT references and translating the amino acid sequences in between those residues.

### Motif analysis.

CDR3β amino acid sequences were trimmed to the region most likely to be in contact with antigenic peptide (IMGT positions 106–117), as the stem positions of CDR3βs are not predicted to be involved with antigen binding (2). The trimmed sequences were then broken into all possible continuous n-mers of size 3, 4, and 5 amino acids. In addition, discontinuous motifs were generated of size 4 and 5, allowing for gaps in the sequences so long as there were still 3 conserved residues. The proportion of each n-mer in the ps-CDR3s was calculated by dividing the number of reads containing that n-mer by the total number of ps-CDR3 reads. To determine fold enrichment, this proportion of ps-CDR3 reads with a given n-mer was divided by the proportion of reads in the resting CDR3βs with the n-mer.

### Network analysis.

Each unique ps-CDR3 sequence was represented as a node and edges between nodes were made if there was a Levenshtein distance of 1 between them or if they shared an enriched motif. Levenshtein distances were determined using the R package *stringdist* (version 0.9.5.1). A self-edge was created on a node for every additional nucleotide sequence that corresponded to the same amino acid sequence. Network object (gml) files were created in R using the *igraph* package (version 1.0.1), and network visualization was performed with Cytoscape 3.7.0, using a force-directed open-CL layout. To evaluate the amount of structure present among ps-CDR3s, the number of edges in ps-CDR3s was compared with the median number of edges in 100 equal-sized random resamplings of resting and activated CDR3β sequences, by creating edges between the sequences of each resampling if they were within a Levenshtein distance of 1, had an enriched motif, or if sequences demonstrated convergence.

### T cell subset sorts and probing for ps-CDR3 sequences.

Cryopreserved PBMCs from 8 of the 27 individuals (Supplemental Table 1) were thawed, and memory CD4^+^ T cells were isolated with the EasySep human memory CD4^+^ T cell enrichment kit. Memory T cells were labeled with APC-Cy7–conjugated anti-CD4, PerCP-Cy5.5–conjugated anti-CRTH2 (clone BM16; BD Biosciences), BV605-conjugated anti-CCR4 (L291H4; BioLegend), AF647-conjugated anti-CXCR5 (J252D4; BioLegend), PE-Cy7–conjugated anti-PD1 (eBioJ105; eBioscience), FITC-conjugated anti-CXCR3 (G025H7; BioLegend), BUV395-conjugated anti-CCR5 (2D7/CCR5; BD Biosciences), eFluor450-conjugated anti-CD161 (HP-3G10; eBioscience), BV711-conjugated anti-CCR6 (11A9; BD Biosciences), and Live/Dead Fixable Blue stain. Live CD4^+^CXCR3^+^CCR5^+^ Th1 cells, CRTH2^+^CCR4^+^ Th2 cells, CD161^+^CCR6^+^ Th17 cells, and CXCR5^+^ Tfh cells were sorted with a FACSAria Fusion instrument. Th2 cells were gated out first, followed by Tfh cells, then Th1 cells, and finally Th17 cells. Sorted T cell subsets then underwent the TCRβ-sequencing protocol described above. These T cell subset libraries were probed for the ps-CDR3 amino acid sequences from the corresponding individuals. To determine the degree of overlap between the ps-CDR3s detected in each subset, a Jaccard index was calculated among the unique ps-CDR3 sequences found in each pairwise combination of subsets. The Jaccard index was calculated using the R package *OmicsMarkeR* (version 1.16.0), and the resulting matrix was visualized with the R package *ComplexHeatmap* (version 2.0.0; ref. 36).

### Data availability.

The TCRβ-sequencing data sets used for this project have been deposited at the National Center for Biotechnology Information database of Genotypes and Phenotypes under accession phs001897.v2.p1.

### Code availability.

The source code used in this study will be available at https://github.com/nealpsmith/peanut_pscdr3 (branch name: Master and commit ID: 068f348cd15ef1ab3360932164a01ba7c120753c).

### Statistics.

A *P* value less than 0.05 was considered significant for this study. In all box plots shown in this manuscript, the lower and upper hinges correspond to the first and third quartiles (the 25th and 75th percentiles). The upper whisker extends from the hinge to the largest value no further than 1.5 × IQR from the hinge (where IQR is the distance between the first and third quartiles). The lower whisker extends from the hinge to the smallest value at most 1.5 × IQR from the hinge. Data beyond the end of the whiskers (“outlying” points) were plotted individually. All comparisons made with matched pairs of values from individuals were tested with a Wilcoxon matched-pairs signed-rank test. Such comparisons include frequencies of CD154^+^ T cells per million CD4^+^ T cells in cultures and differences in the ratios of diversities in resting, activated, and ps-CDR3 pools. Evaluation of enrichment of ps-CDR3s at minimum Hamming/Levenshtein distances utilized a 2-sided Fisher’s exact test, comparing the median frequencies of activated or resting CDR3βs to the observed frequency in the ps-CDR3s. To assess differences in diversity, the empirical cumulative distribution function of bootstrap delta distribution was used as described by Stern et al. (37). Comparisons of public and private ps-CDR3 repertoire features, such as the number of nucleotide insertions, total rearrangements, and node degree in the ps-CDR3 network, were done with a Mann-Whitney *U* test. A 2-sided Fisher’s exact test was used to compare the proportion of public ps-CDR3s with an enriched motif to private ps-CDR3s with a motif. Similarly, a 2-sided Fisher’s exact test was used to compare the proportion of public ps-CDR3s with an edge in the ps-CDR3 network to that of the private ps-CDR3s. A *Z* test was used to compare the number of edges created within the ps-CDR3 network to that of the distribution of edges created by 100 equal-sized random samplings of resting or activated CDR3βs. Associations between CD4^+^ T cell subset ps-CDR3s and clonal expansion were evaluated with logistic regression using the *glm* function in R. For each subset, ps-CDR3 membership was predicted with the sequences’ read counts.

### Study approval.

All participants were recruited with informed consent prior to sample collection, and the study was approved by the Institutional Review Board of Partners Healthcare (Boston, Massachusetts, USA) (protocol no. 2012P002153). The study was conducted according to the principles of the Declaration of Helsinki.

## Author contributions

NPS, BR, WGS, and JCL designed the study. NPS and BR performed all of the lab work and generated all of the bulk TCR-sequencing data. AAT and BM generated the single-cell TCR-sequencing data. NPS, YVV, and WGS performed the data analysis. NPS and BR wrote the manuscript. JJM developed the pMHC tetramers used in this study. All authors reviewed and approved the final version of the manuscript.

## Supplementary Material

Supplemental data

## Figures and Tables

**Table 1:**
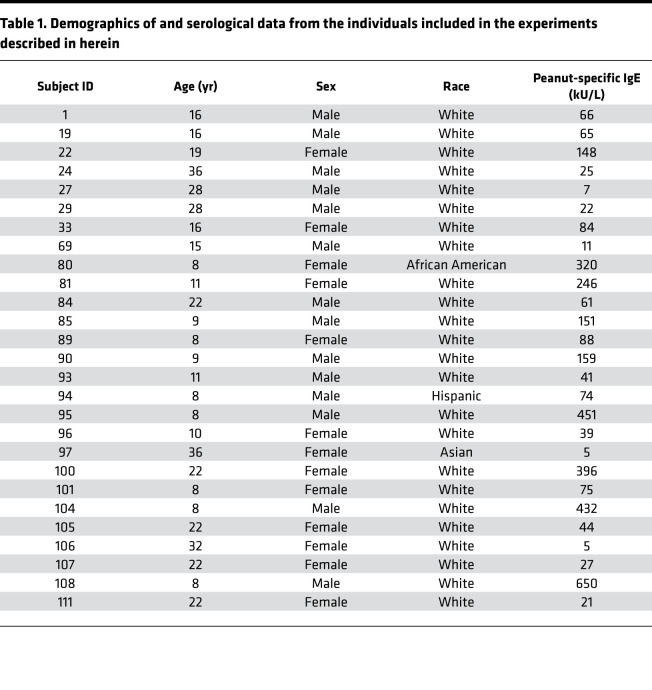
Demographics of and serological data from the individuals included in the experiments described in herein

**Table 2:**
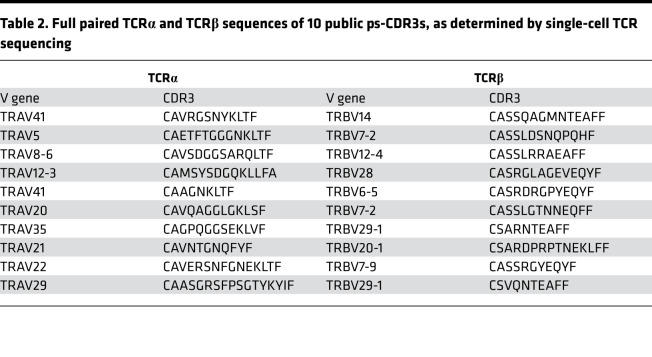
Full paired TCRα and TCRβ sequences of 10 public ps-CDR3s, as determined by single-cell TCR sequencing

**Table 3:**
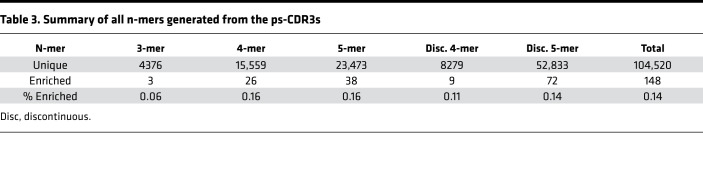
Summary of all n-mers generated from the ps-CDR3s

## Version 1 05/25/2021

In-Press Preview

## Version 2 07/08/2021

Electronic publication
