# Supplementary material for: Identification of antigen-specific TCR sequences based on biological and statistical enrichment in unselected individuals
Source: JCI Insight. 2021 Jul 8;6(13):e140028. doi: 10.1172/jci.insight.140028 (PMC8410028; doi:10.1172/jci.insight.140028)
Supplement: Supplemental data [file jciinsight-6-140028-s096.pdf]

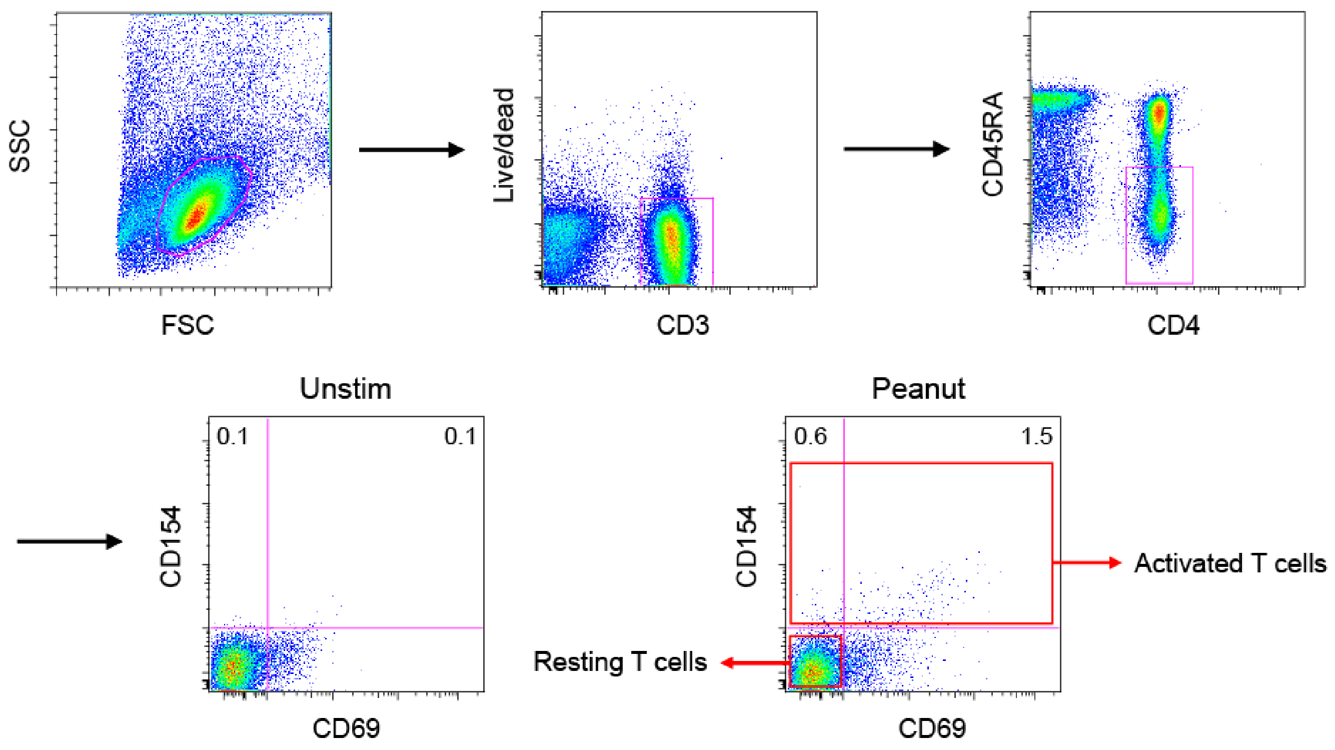

Supplemental figure 1. FACS gating strategy for isolating peanut-activated memory CD4^{+} T cells (CD154^{+}) and resting memory CD4^{+} T cells (CD154^{-}CD69^{-}) from the PBMCs of peanut-allergic individuals.

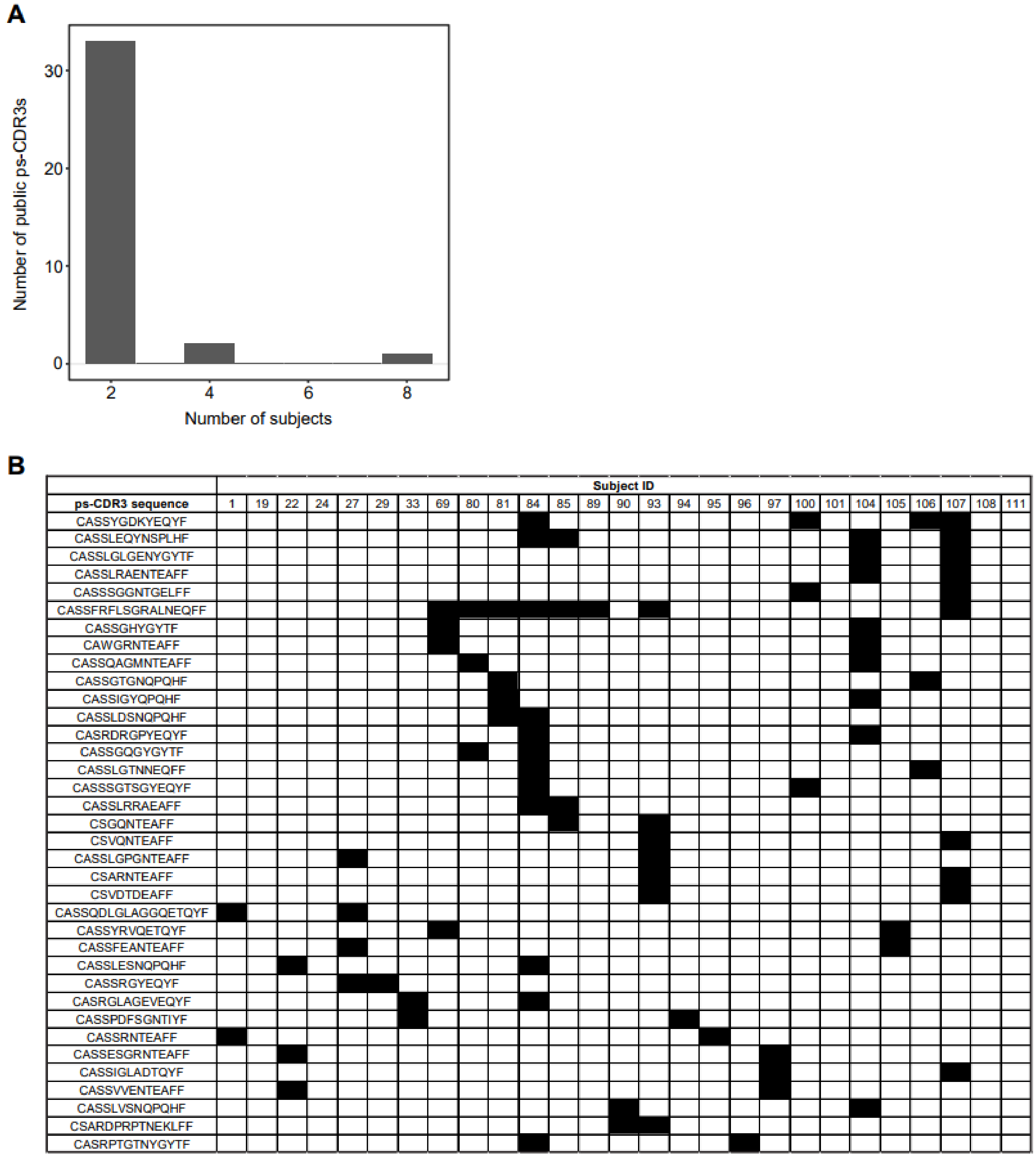

Supplemental Figure 2: Distribution of public ps-CDR3s across the peanut-allergic subjects. A, Distribution of the public ps-CDR3s over the subjects. Shown is the number of public ps-CDR3s present in a given number of individuals. B, Summary of the presence of each unique public ps-CDR3 in each subject.

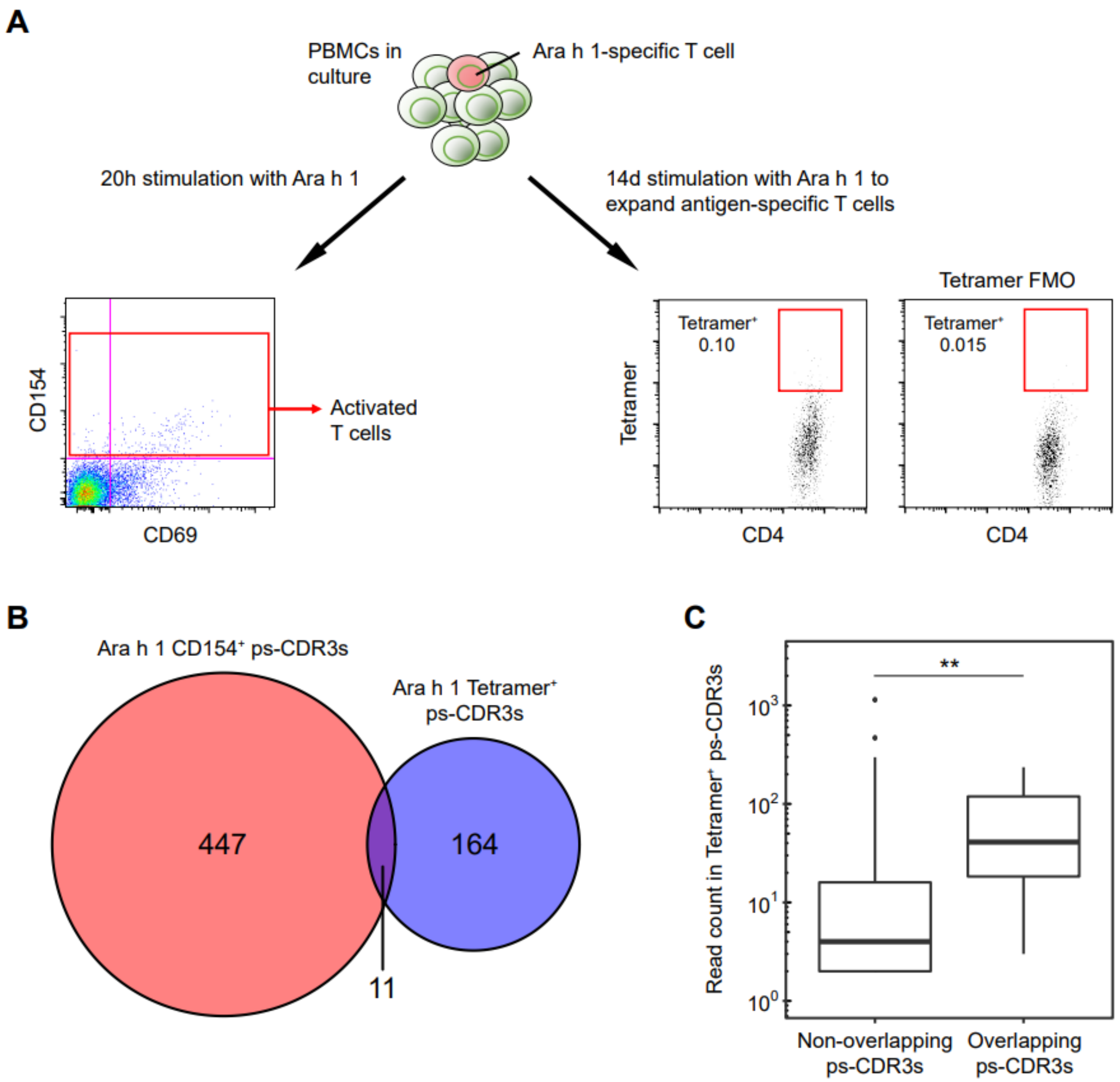

Supplemental Figure 3: ps-CDR3s from Ara h 1 tetramer^{+} T cells are present among ps-CDR3s derived from Ara h 1-activated T cells, and are highly expanded. A, General schema of the experimental approach. PBMCs from subject 107 were stimulated with the major peanut allergen Ara h 1 (50 µg/ml) for 20 hours, and ps-CDR3s were derived from CD154^{+} T cells. In addition, PBMCs from the same subject were cultured for 2 weeks with Ara h 1, and ps-CDR3s were derived from sorted Ara h 1 (DRB1*03:01, AA 415-425) tetramer^{+} T cells (see Methods). FMO = Fluorescence-minus-one control. B, Eleven ps-CDR3s from Ara h 1 tetramer^{+} T cells were also present in the ps-CDR3 pool from Ara h 1-activated CD154^{+} T cells. C, The 11 overlapping ps-CDR3s were more clonally expanded than the other ps-CDR3s derived from Ara h 1 tetramer^{+} T cells. Boxplots represent distributions of read counts of the corresponding ps-CDR3 sequences (** $P < 0.01$, Mann-Whitney U test).

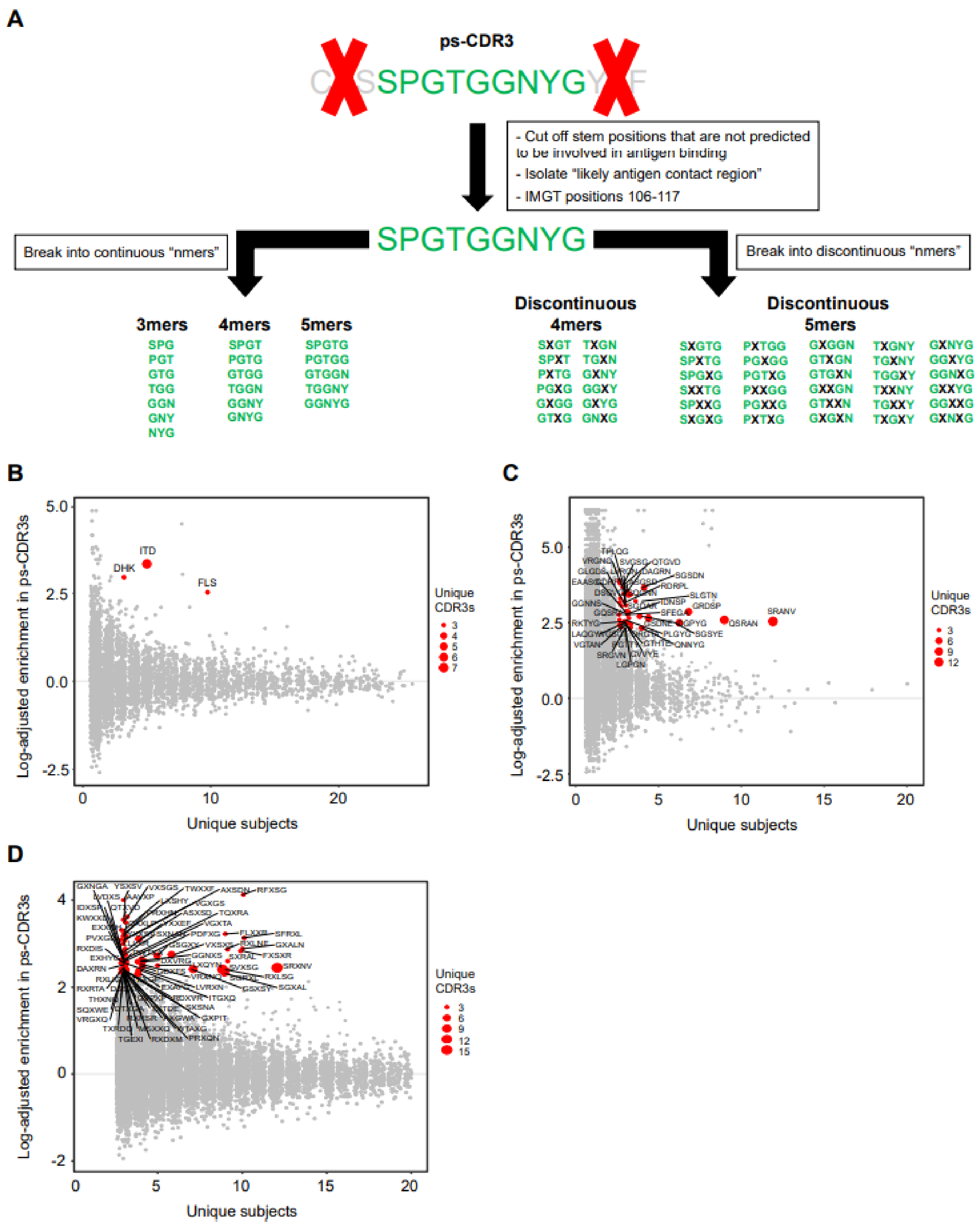

Supplemental Figure 4: Motif analysis of ps-CDR3s. A, Schema of nmer generation with an example ps-CDR3 (see Methods). B-D, The ps-CDR3s contain shared and enriched 3-mer, 5-mer, and discontinuous 5-mer motifs as compared to resting CDR3s. Red points represent the most dominant motifs, which were ≥ 10-fold enriched, in ≥ 3 unique ps-CDR3s derived from ≥ 3 subjects.

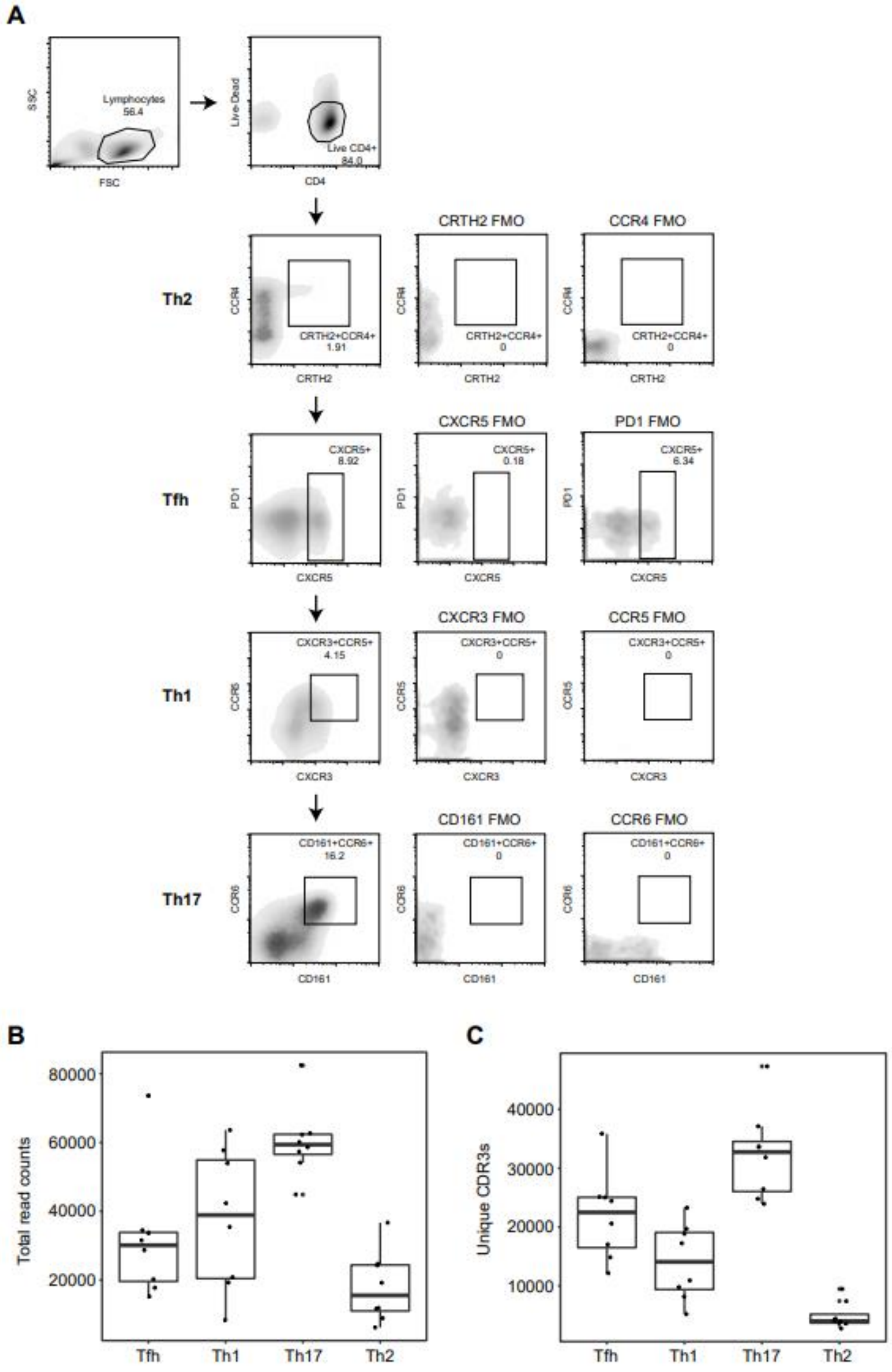

Supplemental Figure 5: Sorting of T cell subsets from peanut-allergic individuals. A, FACS gating strategy for isolating bulk memory $CD4^{+}$ $T_{H}2$ ($CRTH2^{+}CCR4^{+}$), T_{FH} ($CXCR5^{+}$), $T_{H}1$ ($CXCR3^{+}CCR5^{+}$), and $T_{H}17$ ($CD161^{+}CCR6^{+}$) cells (see Methods). FMO = Fluorescence-minus-one control. B, Distribution of the total read counts in the T cell subset samples. C, Distribution of unique CDR3β sequences in the T cell subset samples.

Supplemental Table 1: Subjects included in each set of experiments described in this paper.

Subject ID	TCRβ-seq CD154+/CD154-	T cell subset sorting	scTCR-seq CD154+	pMHC-Tetramer TCRβ-seq
1	X			
19	X			
22	X	X		
24	X			
27	X	X		
29	X	X		
33	X	X	X	
69	X		X	
80	X			
81	X	X		
84	X		X	
85	X			
89	X			
90	X		X	
93	X		X	
94	X			
95	X		X	
96	X		X	
97	X	X	X	
100	X			
101	X			
104	X			
105	X		X	
106	X	X	X	
107	X	X	X	X
108	X			
111	X		X	

Supplemental Table 2: HLA genotypes of the subjects in this study.

Subject ID	DPA1_allele1	DPA1_allele2	DPB1_allele1	DPB1_allele2	DQA1_allele1	DQA1_allele2	DQB1_allele1	DQB1_allele2	DRB1_allele1	DRB1_allele2	DRB3_allele1	DRB3_allele2	DRB4	DRB5
1	1:03:01	2:02:02	1:01:01	3:01:01	1:03:01	3:03:01 AM	3:01:01	6:03:01	13:01:01	4:01:01	1:01:02		1:03:01	
19	2:01:01	2:07:01	11:01:01	19:01:01	1:03:01	2:01:01	2:02:01	6:03:01	13:01:01	7:01:01	2:02:01		1:01:01	
22	1:03:01	1:03:01	4:01:01	4:01:01	1:02:01	3:01:01	3:02:01	6:02:01	15:01:01	4:02:01			1:03:01	1:01:01
24	2:01:01	2:01:02	1:01:01	17:01:01	2:01:01	5:01:01	2:01:01	2:02:01	3:01:01	7:01:01	1:01:02		1:01:01	
27	1:03:01	2:01:01	3:01:01	10:01:01	1:04:01	3:01:01	3:02:01	5:03:01	14:54:01	4:01:01	2:02:01		1:03:01	
29	1:03:01	2:01:01	4:01:01	13:FNVU	3:01:01	5:05:01	3:01:01	3:02:01	11:04:01	4:02:01	2:02:01		1:03:01	
33	1:03:01	2:01:07	4:01:01	13:FNVU	1:02:01	3:02:01	3:03:02	6:09:01	13:02:01	09:CWA	3:01:01		1:03:02	
69	1:03:01	1:03:01	2:01:02	13:FNVU	1:02:01	2:01:01	2:02:01	6:02:01	15:01:01	7:01:01			1:01:01	1:01:01
80	1:03:01	2	4:01:01	85:01:01	2:01:01	5:01:01	2:01:01	2:02:01	3:01:01	7:01:01	1:01:01		1:03:01	
81	1:03:01	1:03:01	2:01:01	4:01:01	1:02:01	3:01:01	3:02:01	6:02:01	15:01:01	4:02:01			1:03:01	1:01:01
84	1:03:01	2:01:01	4:01:01	10:01:01	1:01:02	1:02:01	5:01:01	6:09:01	1:02:01	13:02:01	3:01:01	3:01:01		
85	1:03:01	1:03:01	4:01:01	4:02:01	5:01:01	5:01:01	2:01:01	2:01:01	3:01:01	3:01:01	1:01:02	1:01:02		
89	1:03:01	2:06	4:02:01	5:01:01	1:02:01	1:02:01	6:02:01	6:02:01	15:01:01	15:01:01				1:01:01
90	1:03:01	1:03:01	4:01:01	4:01:01	5:01:01	5:05:01	2:01:01	3:01:01	3:01	11:01	1:01:02	2:02:01		
93			4:01:01	4:01:01	1:04:01	5:01:01	2:01:01	5:03:01	3:01:01	14:01:01	1:01:02	2:24		
94	1:03:01	1:03:01	2:01:02	4:01:01	1:01:01	1:02:01	5:01:01	6:02:01	1:03:01	15:01:01				1:01:01
95	1:03:01	1:03:01	4:01:01	4:01:01	1:02:01	5:05:01	3:01:01	6:02:01	15:01:01	11:01:01	2:02:01			1:01:01
96	1:03:01	2:01:02	1:01:01	2:01:02	3:03:01	5:05:01	3:01:01	3:02:01	12:JV	4:01:01	2:02:01		1:03:01	
97	1:03:01	1:03:01	2:01:02	48:01:00	3:01:01	5:03:01	3:01:01	3:02:01	13:12:01	4:03:01	2:02:01		1:03:01	
100	1:03:01	1:03:01	2:01:02	4:01:01	5:05:01	5:05:01	3:01:01	3:01:01	11:04:01	11:04:01	2:02:01	2:02:01		
101	1:03:01	1:03:01	3:01:01	4:01:01	1:02:01	1:02:01	6:02:01	6:04:01	15:01:01	13:02:01	3:01:01			1:01:01
104	1:03:01	1:03:01	4:01:01	4:01:01	1:02:01	2:01:01	3:03:02	6:02:01	15:01:01	7:01:01			01:03:01 N	1:01:01
105	1:03:01	2:01:01	2:01:02	5:01:01	3:01:01	5:01:01	2:01:01	3:02:01	3:01:01	4:02:01	1:01:02		1:03:01	
106	1:03:01	1:03:01	4:01:01	104:01:01	1:01:02	2:01:01	3:01:01	5:01:01	1:02:01	7:01:01			1:01:01	
107	1:03:01	1:03:01	4:01:01	20:01:01	3:01:01	5:01:01	2:01:01	3:02:01	3:01:01	4:04:01	1:01:02		1:03:01	
108	1:03:01	1:03:01	3:01:01	4:02:01	1:02:01	5:01:01	2:01:01	6:04:01	3:01:01	13:02:01	1:01:02	3:01:01		
111	1:03:01	1:03:01	2:01:02	4:01:01	1:03:01	5:05:01	3:01:01	6:03:01	13:01:01	13:05:01	1:01:02	2:02:01		
